# The importance of workplace exercise

**DOI:** 10.47626/1679-4435-2021-666

**Published:** 2021-12-30

**Authors:** Victor Matheus Lopes Martinez

**Affiliations:** 1 Programa de Pós-Graduação em Psicologia, Pontifícia Universidade Católica do Rio Grande do Sul, Porto Alegre, RS, Brazil.

**Keywords:** workplace exercise, RSIs/WMSDs, occupational stress, burnout syndrome, ginástica laboral, LERs/DORTs, estresse ocupacional, síndrome de burnout

## Abstract

This narrative review demonstrates the importance of workplace exercise for workers’ quality of life and productivity and the reduced number of sick leaves due to repetitive strain injuries/work-related musculoskeletal disorders. To this end, PubMed, SciELO, and CAPES databases were searched, as well as books on the topic. The general objective of this article is to elucidate and disseminate the importance of workplace exercise programs for workers’ health and work performance. Data collection revealed that workplace exercise directly improves workers’ quality of life, reducing the rates of repetitive strain injuries/work-related musculoskeletal disorders, occupational stress, and burnout syndrome. In conclusion, the implementation of a workplace exercise program is of great value for both workers and the company.

## Resumo

Este artigo trata sobre a importância da ginástica laboral em relação à qualidade de vida e produtividade dos trabalhadores e à diminuição do número de afastamentos oriundos de lesões por esforço repetitivo/distúrbios osteomusculares relacionados ao trabalho. Para isso, foi realizada uma pesquisa bibliográfica de cunho narrativo nas bases eletrônicas PubMed, SciELO e Capes e em livros sobre o tema. O estudo em questão teve como objetivo geral esclarecer e disseminar a importância da existência de um programa de ginástica laboral para a saúde dos colaboradores e o rendimento laboral. A coleta dos dados revelou que a ginástica laboral incide diretamente na melhora da qualidade de vida dos funcionários, diminuindo os índices de lesões por esforço repetitivo/distúrbios osteomusculares relacionados ao trabalho, estresse ocupacional e síndrome de *burnout.* Conclui-se que a aplicabilidade de um programa de ginástica laboral é de extrema importância tanto para os funcionários quanto para a empresa.

## INTRODUCTION

The Brazilian Ministry of Health has recently emphasized the importance of workplace exercise by showing alarming data: in Brazil, a mean of 100,000 workers per year are on sick leave due to symptoms of work-related musculoskeletal disorders (WMSDs), and companies spend approximately R$89,000 per sick worker anually.^1^ These are the problems that, in a financially attractive and less evasive way, workplace exercise aims to solve. Workplace exercise is a tool that aims to improve working conditions and quality of life through a physical exercise program including mobility, stretching, and strengthening exercises adapted to work-related activities. It may reduce fatigue and, consequently, optimize workers’ productivity and satisfaction.^2^

My personal and professional experience as a postural and workplace exercise instructor for 2 years at the Universidade do Vale do Rio dos Sinos (UNISINOS) and as a workplace exercise intern at the Federal Court of Accounts of the State of Rio Grande do Sul (TCE/RS), where I heard workers complain about the harms caused by the lack of regular physical exercise and point out the benefits they gained from exercise programs, encouraged me to write this literature review as a way to illustrate and elucidate the current knowledge and benefits provided by workplace exercise to workers and the work environment.

Therefore, the general objective of this study is to elucidate the importance of a workplace exercise program for workers’ health and work performance. The relevance of this study lies in understanding and promoting the benefits of workplace exercise, as we are facing a pandemic that has led many employees to work remotely from home without optimal ergonomic conditions, which could cause or further aggravate repetitive strain injuries (RSIs) and WMSDs. To this end, PubMed, SciELO, and CAPES electronic databases were searched, as well as books on the following topics: workplace exercise, RSIs/WMSDs, and stress.

### WORKPLACE EXERCISE: CONCEPTS AND DEFINITIONS

According to the Regional Council of Physical Education of the 9th Region of the State of Paraná (Conselho Regional de Educação Física da 9ª Região Estado do Paraná), workplace exercise is a type of physical activity conducted at the workplace that is mostly focused on occupational health. It is planned and conducted by physical education professionals during working hours, consisting of physical exercises that aim to improve workers’ health according to their specific job function.^3^

Workplace exercise is a type of physical activity that is planned and conducted at the workplace and aims to create spaces where workers can spontaneously take a break from their repetitive routine to exercise their body and mind and further develop self-knowledge, which may improve their relationship with the work environment. Therefore, workplace exercise improves workers’ productivity and quality of life.^4^ According to the Federal Council of Physical Education, workplace exercise is a set of exercises that seek to compensate the physical and mental efforts required by specific job functions. In addition, it allows workers to take an active break from their routine in the organizational environment.^5^

Workplace exercise, as the term suggests, is a set of physical exercises performed at the workplace. Workplace exercise programs aim to prevent the onset of RSIs and WMSDs through articular and muscular mobility exercises, promoting physical benefits, reducing range of motion loss, and providing a space within the work environment where workers can socialize and take a break while they exercise their body and mind. Therefore, these gains in quality of life will consequently lead to increased productivity and reduced rates of sick leave due to injuries.

Within this context, we understand that workplace exercise programs consist of physical activities adapted to the organizational environment with the aim of providing an active break before, during, and/or after working hours. Its main objective is to reduce the rates of RSIs, WMSDs, and occupational stress through articular mobility and muscle stretching exercises, reducing range of motion loss, injuries, and/or work-related disorders. It also provides a space for workers to socialize and take a break from their routine.

Workplace exercise is a sick-leave prevention tool that improves organizational climate and workers’ quality of life, thus increasing employee productivity and satisfaction. It consists, therefore, of exercises that are planned, guided, and conducted by physical education professionals with the goal of collective health prevention and/or promotion.

### TYPES OF WORKPLACE EXERCISE

There is more than one type of workplace exercise, and definitions vary according to different authors. There are five main types^4^:

#### 1) Preparatory workplace exercise

In this modality, workers exercise before their work shift (beginning of the morning, afternoon, or night). It may be used as a morning “awakening”, especially for workers who handle tools and instruments with a risk of accident due to human error.

#### 2) Compensatory workplace exercise

Also known as break exercise or active break, it is the most commonly implemented modality. In this modality, workers exercise during working hours or at peak fatigue time, usually 3 to 4 hours after the beginning of their shift. Compensatory workplace exercise aims to prevent poor posture when performing routine or work activities. It includes exercises that stretch the muscles used during the workday (agonists) and strengthens the muscles less used during routine tasks (antagonists).

#### 3) Relaxing workplace exercise

In this modality, workers exercise at the end of the work shift, usually 10 to 15 minutes before the shift ends. It is recommended for those who have direct contact with the public, such as bankers and customer service workers. These workers need relaxation due to the stress accumulated during the day; they may receive massages in the dorsal, cervical, and lumbar segments, shoulders, feet, or in other painful regions.

#### 4) Corrective workplace exercise

The objectives of this modality are to correct posture and restore any muscle or joint imbalances. It includes specific physical exercises to stretch shortened muscles (the ones often used during work activities) and strengthen the weakened ones (less used). To this end, a specific group of workers with the same complaints (10 to 12 people) is selected to participate in a common workplace exercise class focused on the group’s complaints.

#### 5) Maintenance workplace exercise

This modality aims to restore workers’ morphophysiological balance to prevent or avoid the onset of chronic degenerative disorders. Workers may engage in exercise before the beginning of the work shift, during lunch break, after the work shift is over, or during the opposite shift. Classes last approximately 30 to 60 minutes, and they may be divided into short sessions of 10, 15, or 20 minutes throughout the day. This modality consists mostly of aerobic exercises, which increase respiratory capacity, and focused exercises for muscle mass gain in specific regions of the body, factors that improve and promote the workers’ daily well-being.

However, some authors believe there are only three main types of workplace exercise programs, which are based on the time they are conducted: preparatory (before the beginning of the work shift), compensatory (during working hours, as an “active break”), and relaxing (at the last minutes of or after the work shift is over).^6,7^

In addition to the aforementioned types, a study described a unicist workplace exercise program. According to the authors, it aims to exercise the body as a whole, focusing on the upper extremities, which are the most affected regions by WMSDs. This modality also includes exercises focused on the lower extremities and the cervical and lumbar segments, in addition to promoting postural guidance and interpersonal relationships before, during, or after working hours.^8^

Therefore, a wide range of workplace exercise programs is hypothesized. Despite small structural differences in applicability methodologies, they all converge toward the same objective: to improve and/or maintain workers’ physical and mental health.

### SCIENTIFIC EVIDENCE FOR WORKPLACE EXERCISE PROGRAMS

As previously described, the main purpose of a workplace exercise program is to promote improved well-being in the work environment by increasing employees’ quality of life and preventing injuries and the onset of psychosomatic disorders. Therefore, it is natural to investigate the actual benefits provided by these programs.

Previous studies have identified several improvements with regular workplace exercise. A study on flexibility, in which hospital workers were assessed after the implementation of a workplace exercise program, compared participants’ flexibility levels at three moments: (1) before the intervention; (2) after the intervention; and (3) 6 months after the intervention. The data showed that, in addition to the benefits observed when comparing the first two time points, flexibility levels at time point 3 remained the same as those at time point 2. This means that there was no significant loss of gained flexibility after the end of the intervention.^9^

Concomitantly, another study also demonstrated increased flexibility in workers who adhered to workplace exercise. In this case, the company already had a workplace exercise program, and the authors conducted a comparative analysis between workers who did and did not adhere to the program. A greater range of motion was observed in employees who engaged in workplace exercise.^10^

In the same year, Martins et al.^11^ published a study referring to the benefits provided by regular workplace exercise. The study aimed to identify whether workplace exercise programs improved flexibility and reduced musculoskeletal pain in workers. Physical activity and musculoskeletal symptom questionnaires were applied to assess the strength of stockroom and administrative workers before and after 6 months of workplace exercise. The results showed a significant decrease in musculoskeletal pain in workers of both departments, as well as improved flexibility in the cervical, trunk, and shoulder regions.^11^

A study conducted at an industrial waste treatment company identified a reduction of more than 50% in sick leaves after the implementation of a workplace exercise program, especially due to musculoskeletal disorders.^12^ The evidence provided by these studies clearly corroborates that workplace exercise programs exponentially improve employees’ health, thus increasing work satisfaction and, therefore, productivity.

### “OCCUPATIONAL” DISORDERS AND WORKPLACE EXERCISE

As previously described, one of the main objectives of workplace exercise is to prevent the onset of RSIs/WMSDs. RSIs/WMSDs are similar but not identical terms, although both refer to the harms caused by uninterrupted work in the work environment. RSIs is the most commonly used term, but it does not comprehend other causes of occupational disorders besides continuous and repetitive strain injuries. This misunderstanding prevents the resolution of problems related to these disorders because it does not address important aspects such as poor posture and inappropriate ergonomics in the workplace.^13^

The Occupational Rheumatology Commission of the Brazilian Society of Rheumatology, as well as other authors, explained that the term “RSIs” has been replaced by “WMSDs”, which goes one step further and implies that occupational disorders are related to the work environment as a whole, ie, they are beyond mechanical factors. Therefore, WMSDs have several causes, including poor posture, stress, organizational climate (dissatisfaction at work), inappropriate office furniture, and personal, social, family, and economic problems, as well as repetitive movements.^13,14^ Importantly, WMSDs only occur when the causal factor is work-related RSIs. Therefore, WMSDs are only caused by work-related injuries, whereas RSIs may occur in any context in which repetitive movements are performed without due care.

According to the Brazilian Ministry of Health, RSIs and WMSDs have increased approximately 184% in the last 10 years (from 2007 to 2016). This is alarming, as it directly affects workers’ productivity, being detrimental to their physical and social well-being. Ultimately, it may result in sick leave and expenses with treatments and indemnities.^1^ Considering the data from the previous studies, RSIs/WMSDs are a problem that affects workers from several departments and business branches. These conditions should always be taken seriously as, besides physical injuries, there may be negative impacts on mental health, such as occupational stress.

Stress is considered the disorder of the 21st century and is defined as a state of tension that ruptures the inner balance of the body. In its early stage, stress is identified as a series of psychosomatic signs and symptoms, such as tachycardia, gastritis, cardiovascular changes, and insomnia, among others.^15^ Stress has spread extensively in the work environment - known as occupational stress - and is linked to excessive stimuli in the workplace, especially when the demands exceed workers’ coping capabilities. Thus, it affects workers’ quality of life and eventually leads to sick leave.^16^

A stress phenomenon, therefore, is based on the person’s self-perception of an event as stressful or not. The way the person reacts to the event is what makes it harmful, and cognitive function is of great importance in this respect. Thus, occupational stress is characterized by negative responses to stimuli in the workplace.^17^

This condition may lead to other disorders, such as burnout syndrome (BS). According to the International Classification of Diseases - 11th Revision (ICD-11), BS is a psychic disorder caused by emotional and physical tension due to working conditions. It causes chronic stress, which in turn may result in exhaustion, distancing, and reduced productivity in the workplace.^18^

In conclusion, the aforementioned disorders are extremely detrimental to workers’ health, productivity, and performance in the occupational setting. RSIs/WMSDs, occupational stress, and BS are some of the disorders caused by work-related activities and may be avoided by workplace exercise programs, as demonstrated by the available scientific evidence.^4^

## CONCLUSIONS

Based on the evidence of the importance and benefits of workplace exercise for occupational health, we can state that, when applied systematically before, during, or after working hours (according to department and work activity), workplace exercise is clearly essential to improve employee attention in the workplace. It helps to avoid unnecessary movements that may result in RSIs or WMSDS, as well as procedural errors that may also cause injuries. In addition, it entails a significant improvement in cognitive aspects related to the mental health of employees, preventing the onset of disorders such as occupational stress and BS. Adherence to and/or implementation of a workplace exercise program is of great value both for employees, who will improve their quality of life, and for the company, given that workers will be more satisfied. This could eventually lead to increased productivity and reduced costs with sick leaves.
